# Effectiveness of Public Health Digital Surveillance Systems for Infectious Disease Prevention and Control at Mass Gatherings: Systematic Review

**DOI:** 10.2196/44649

**Published:** 2023-05-19

**Authors:** Noha Maddah, Arpana Verma, Maryam Almashmoum, John Ainsworth

**Affiliations:** 1 Division of Informatics Imaging and Data Sciences, School of Health Sciences, Faculty of Biology, Medicine and Health, Manchester Academic Health Science Centre Centre for Health Informatics The University of Manchester Manchester United Kingdom; 2 Department of Health Services and Hospitals Administration, Faculty of Economics and Administration King Abdulaziz University Jeddah Saudi Arabia; 3 Division of Population Health, Health Services Research & Primary Care, Faculty of Biology, Medicine and Health, Manchester Academic Health Sciences Centre The University of Manchester Manchester United Kingdom; 4 Nuclear Medicine Department, Faisal Sultan Bin Eissa, Kuwait Cancer Control Center Kuwait Kuwait

**Keywords:** public health, digital surveillance system, infectious disease prevention and control, mass gathering event, systematic review

## Abstract

**Background:**

Mass gatherings (MGs; eg, religious, sporting, musical, sociocultural, and other occasions that draw large crowds) pose public health challenges and concerns related to global health. A leading global concern regarding MGs is the possible importation and exportation of infectious diseases as they spread from the attendees to the general population, resulting in epidemic outbreaks. Governments and health authorities use technological interventions to support public health surveillance and prevent and control infectious diseases.

**Objective:**

This study aims to review the evidence on the effectiveness of public health digital surveillance systems for infectious disease prevention and control at MG events.

**Methods:**

A systematic literature search was conducted in January 2022 using the Ovid MEDLINE, Embase, CINAHL, and Scopus databases to examine relevant articles published in English up to January 2022. Interventional studies describing or evaluating the effectiveness of public health digital surveillance systems for infectious disease prevention and control at MGs were included in the analysis. Owing to the lack of appraisal tools for interventional studies describing and evaluating public health digital surveillance systems at MGs, a critical appraisal tool was developed and used to assess the quality of the included studies.

**Results:**

In total, 8 articles were included in the review, and 3 types of MGs were identified: religious (the Hajj and Prayagraj Kumbh), sporting (the Olympic and Paralympic Games, the Federation International Football Association World Cup, and the Micronesian Games), and cultural (the Festival of Pacific Arts) events. In total, 88% (7/8) of the studies described surveillance systems implemented at MG events, and 12% (1/8) of the studies described and evaluated an enhanced surveillance system that was implemented for an event. In total, 4 studies reported the implementation of a surveillance system: 2 (50%) described the enhancement of the system that was implemented for an event, 1 (25%) reported a pilot implementation of a surveillance system, and 1 (25%) reported an evaluation of an enhanced system. The types of systems investigated were 2 syndromic, 1 participatory, 1 syndromic and event-based, 1 indicator- and event-based, and 1 event-based surveillance system. In total, 62% (5/8) of the studies reported timeliness as an outcome generated after implementing or enhancing the system without measuring its effectiveness. Only 12% (1/8) of the studies followed the Centers for Disease Control and Prevention guidelines for evaluating public health surveillance systems and the outcomes of enhanced systems based on the systems’ attributes to measure their effectiveness.

**Conclusions:**

On the basis of the review of the literature and the analysis of the included studies, there is limited evidence of the effectiveness of public health digital surveillance systems for infectious disease prevention and control at MGs because of the absence of evaluation studies.

## Introduction

### Mass Gatherings and the Transmission of Infectious Diseases

Emerging and re-emerging infectious diseases such as HIV and AIDS, severe acute respiratory syndrome (SARS), and influenza continue to pose considerable national and global public health concerns [1] based on reported cases of these viruses leading to outbreaks [2]. They remain a severe public health problem in the 21st century and a leading cause of death worldwide [3]. According to the World Health Organization (WHO), infectious diseases are the second leading cause of death in humans, with approximately 15 million fatalities annually [3,4]. Infectious diseases are the most common health threats associated with religious, sporting, and festival mass gatherings (MGs) [5]. A leading global concern regarding MGs is the spread of infectious diseases and their importation and exportation as they spread from attendees to the general population. This spread causes substantial problems for the host country’s health system [6]. International travel, which has increased and become faster, facilitates the transmission of infectious diseases during MG events from attendees to the host country’s local population and vice versa. Examples of outbreaks include the 2002/2003 SARS pandemic, the 2009 swine influenza pandemic, the 2012 Middle East respiratory syndrome outbreak, the 2014 Ebola epidemic, and the recent COVID-19 pandemic in 2020 [7].

The transmission of an infectious disease poses a high risk for attendees at MGs because of crowd size, density, and the duration of the gathering, especially high-profile events such as sporting events (eg, the Olympic Games and the Federation International Football Association [FIFA] World Cup), religious events (eg, the Hajj and World Youth Day), cultural events (eg, Glastonbury Music Festival), and MGs involving national security (eg, political conventions). Respiratory, zoonotic, vector-borne, fecal, oral, sexual, and blood-borne transmission have been reported to be leading causes of morbidity and mortality worldwide [8].

Countries prepare for MG events by following the WHO 2005 International Health Regulations for public health planning, surveillance, and response during MGs [9]. They are required to develop, strengthen, and maintain the capacity of their health care systems to detect, assess, notify, and report risk events to international public health authorities. Public health authorities ensure preparedness for infectious disease hazards that can spread internationally [10]. They use risk assessments as an essential step in the preparation for MGs along with MG stakeholders to strengthen the capacity of management during an MG, mitigate the risk of infectious disease transmission, and decrease pressure on the health system. Preparedness begins with the identification, evaluation, and prioritization of public health threats [11]. There are several factors event planners consider when using risk assessment tools that contribute to the health and safety of participants: weather, attendance, duration of the event, location of the event, event type, crowd mood, alcohol or drug use, crowd density, and age of attendees [9]. Examples of health risk assessment frameworks and tools are the Sendai Framework 2015-2030 developed by the United Nations, the Jeddah Tool developed in 2016 by the Global Centre for Mass Gathering Medicine, and the WHO COVID-19 Mass Gathering Risk Assessment [11-13].

### Disease Surveillance

Public health surveillance is essential for assessing, predicting, and mitigating infectious disease outbreaks [14]. Surveillance plays a substantial role in preparedness for MGs. It is necessary to provide health decision makers with timely and accurate information about infectious disease events to set priorities, identify the need for interventions, and evaluate their effects [15].

During the last 2 decades, experts have emphasized the importance of strengthening public health surveillance and consider it vital to recognize emerging infectious diseases and track the prevalence of those that are more established [1]. Public health surveillance is defined as “the ongoing systematic collection, analysis, and interpretation of data, closely integrated with the timely dissemination of the resulting information to those responsible for preventing and controlling disease and injury” [15].

An important function of surveillance systems is to monitor infectious diseases that have pandemic potential (eg, SARS and influenza), with timely detection of health events such as outbreaks, especially at MGs [1]. Traditional and disease-specific surveillance relies on passive routine reporting by health care facilities and diagnostic laboratories for structured predefined information regarding infectious disease events. However, this indicator-based surveillance (IBS) is inefficient because of limited resources, time, and reporting systems, which results in the incomplete reporting of data on emerging infectious diseases [16,17].

Other methods of surveillance have emerged with advancements in computational sciences to complement the shortcomings of IBS and improve the timeliness and sensitivity of surveillance systems [17]. Digital surveillance uses the internet, other computer-based systems, and emerging technologies for communications and diagnosis [18]. A description of each type is presented in Textbox 1.

**Types of surveillance systems.**

Traditional or indicator-based surveillance (disease-specific surveillance)

- Surveillance of specific pathogens, diseases, or syndromes in a target population [3]

Digital surveillance

- Attempts to provide knowledge of public health issues by analyzing health information stored digitally, as well as the distribution and patterns governing access to these data [18]

Multiple public health interventions have been used to mitigate the spread of infectious diseases through early detection, prediction, and management (Table 1). A web-based real-time surveillance system (Google Flu) was used to identify regional infectious disease activity using search queries [17]. Monitoring devices and sensors are used for crowd control and tracking, such as wireless sensor networks and radiofrequency identification devices. Epidemic modeling and simulation of mixing patterns and disease transmission use computational modeling tools such as Susceptible-Infected-Recovered and agent-based modeling to represent the dynamics of the disease and simulate the behavior of individuals [19]. Event-based surveillance (EBS) systems and sites such as Health Map, BioCaster, EpiSPIDER, ProMED-mail, and the Global Public Health Intelligence Network are used to detect outbreaks and emerging public health threats [17].

**Table 1:** Technologies used for the prediction, detection, and control of emerging infectious disease threats.

Technology	Description	Application
Google Flu	Web-based real-time surveillance	Real-time monitoring of disease activity (ie, seasonal influenza activity) [17]
Wireless sensor networks and radiofrequency identification devices	Monitoring devices and wireless sensing technology	Electronic wearable sensors for real-time tracking and identification [19]
Agent-based modeling	Infectious disease modeling and simulation	Epidemic simulation assessment of determinants of disease spread; design of containment interventions [17,19]
Susceptible-Infected-Recovered	Infectious disease modeling and simulation	Traditional epidemic modeling [19]
HealthMap, BioCaster, EpiSPIDER, ProMED-mail, and GPHIN^a^	EBS^b^	Outbreak and an emerging public health threat detected (ie, SARS^c^) [17]

^a^GPHIN: Global Public Health Intelligence Network.

^b^EBS: event-based surveillance.

^c^SARS: severe acute respiratory syndrome.

Digital surveillance conducted using either passive or active approaches for data collection has become essential for infectious disease surveillance [16]. Passive surveillance is inexpensive and provides critical information for monitoring community health compared with active surveillance as critical data are reported by multiple health institutions such as hospitals, clinics, and public health units. In contrast, active surveillance is an expensive means of providing timely and accurate information on health conditions collected directly by staff members [20]. Technological advances in communication and unofficial mechanisms such as websites and social media simplify detection and monitoring and improve the response to health problems, thus reducing the potential damage caused by them [21].

Infectious disease prevention and control have been the focus of attention in research for many years, especially with the re-emergence of global pandemics. Studies have investigated different technologies and surveillance system applications that have the potential to detect outbreaks. Some studies have examined big data opportunities for the early detection of disease outbreaks in global MGs by applying different approaches (syndromic surveillance systems, internet data, monitoring devices, and epidemic modeling and simulation) for real-time aggregation and analysis of data from relevant sources [19]. Infectious disease surveillance and control and electronic surveillance network systems used for religious MGs such as the Hajj have also been investigated [6]. A variety of digital technologies used in public health responses to pandemics have been examined, such as social media and web-based searches, wearables and sensors, computer vision, machine learning, digital diagnostic genomics, and visualization tools [22]. They include more advanced tools that provide wireless connectivity in the manufacturing and service sectors to enhance automation, such as Industry 4.0 technologies (known as the Fourth Industrial Revolution) and their applications for fighting global pandemics (eg, COVID-19) [10].

Disease surveillance during MGs has existed for years and has been performed using traditional passive methods. Traditional surveillance systems have been an essential component of public health strategies for many decades, but they are ineffective against emerging infectious diseases. However, digital surveillance can improve the sensitivity and timeliness of health event detection [14,18].

Effective surveillance is achieved through the early identification of public health threats such as emerging infectious diseases. Monitoring and forecasting of emerging and re-emerging infectious diseases is of particular interest [23]. The timely collection, analysis, and interpretation of health data are conducted using appropriate surveillance systems. Thus, an effective and immediate response for disease control at MGs requires enhanced surveillance that informs the health outcomes and exposures to prioritize using risk assessment tools. It also requires the capacity for adequate surveillance to receive and analyze information rapidly [9].

Public health surveillance is critical for managing public health threats during international MGs [24], and it ensures that abnormal events and potential hazards that may threaten attendees’ health are addressed early in the event. Effective planning and responses to infectious disease threats can be achieved using early warning signals provided by an appropriate surveillance system during an MG [25].

### Objectives

This review aimed to identify public health digital surveillance systems that have been implemented for infectious disease prevention and control at MG events and to review evidence of their effectiveness for the prevention and control of infectious diseases at MG events.

## Methods

### Information Sources

A systematic review was conducted in accordance with the PRISMA (Preferred Reporting Items for Systematic Reviews and Meta-Analyses) guidelines [26]. The author systematically searched 4 electronic databases (Ovid MEDLINE, Embase, CINAHL, and Scopus) in January 2022 for relevant articles published in English up to January 2022. A combination of search terms was used based on a framework developed by the author that divided the topic into 4 areas of interest (context: MGs, phenomenon of interest: public health, focus: infectious disease prevention and control, and intervention: digital technologies).

### Search Strategy

The authors combined Medical Subject Heading terms (“Mass Gatherings,” “Public Health,” “Infectious Disease,” and “Digital Technology”) derived from the Ovid MEDLINE and Embase databases with keywords from the relevant literature. The CINAHL and Scopus databases were searched without using subject terms as the data yielded by the search of Ovid MEDLINE and Embase had already been maximized. The authors minimized the search terms used in Scopus owing to differences in how the queries were processed in Scopus compared with the way they were processed in the other databases. The first search in Scopus using the same search terms as those used in Ovid MEDLINE, Embase, and CINAHL yielded numerous results because of the confusion between some of the search terms, such as “Large events,” “Large Gatherings,” “Pandemic,” and “Outbreak.” A description of the search strategies used in the Ovid MEDLINE, Embase, CINAHL, and Scopus databases is presented in Multimedia Appendix 1.

### Eligibility Criteria and the Selection Process

**Figure 1:**
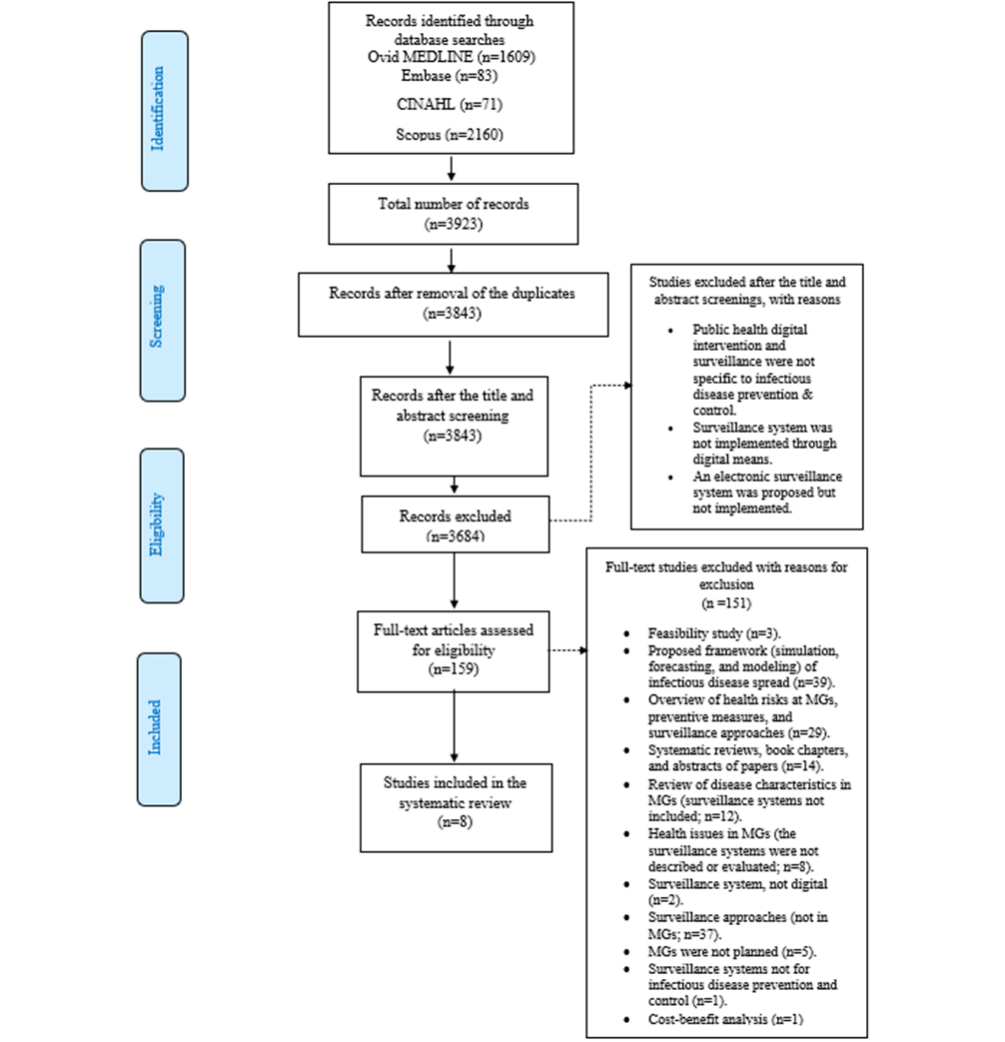
Flow diagram of the selection process for the included studies. MG: mass gathering.

One author (NM) conducted the database search and independently screened the titles of all articles retrieved from the 4 databases to identify eligible studies for full-text review. If the title met the eligibility criteria, the abstract was screened by an additional author (MA) to check whether the study fulfilled the inclusion criteria. Both NM and MA reviewed the full text of the studies with the potential to meet the eligibility criteria and examined their relevance to the topic of this review. The included studies consisted of journal articles published in English up to 2022 that described or evaluated an MG or large religious, sporting, or musical event; implemented an intervention such as disease surveillance during the event using electronic means; and described the effects and outcomes of the intervention. Studies that were published in book chapters or periodic articles were excluded from the analysis, and studies in which the disease surveillance system was implemented in a non-MG setting or the surveillance system in the MG setting was not implemented using electronic means were excluded from the analysis. The author used an additional assessment framework to evaluate the included studies by assessing the characteristics of (1) the MG event and (2) the surveillance system or digital intervention that was implemented. A description of the inclusion and exclusion criteria of the studies included in this review is presented in Table 2. Details of the inclusion and exclusion criteria are shown in Figure 1.

**Table 2:** Inclusion and exclusion criteria of the studies included in this review.

Criteria	Inclusion	Exclusion
Limits	Written in the English language	Non–English-language studies
Type of MG^a^	Planned MG	Unplanned MGs
Type of intervention	A surveillance system implemented in an MG event using an electronic means (electronic, web-based, or digital surveillance system)	Traditional, indicator-based, non–web-based, or nonelectronic means
Type of study	Journal articles and peer-reviewed studies	Book chapters, periodic articles, and reports
Type of surveillance system	Implemented for infectious disease prevention and control at an MG event	Implemented for health hazards; infectious disease surveillance described at a country level not specific to the MG event

^a^MG: mass gathering.

### Data Management

The authors used EndNote (Clarivate Analytics), a reference manager, after the search to record potential studies retrieved and eliminate duplicates of articles across the 4 databases.

### Quality Assessment

Owing to the absence of appraisal tools and criteria appropriate for interventional studies on public health surveillance systems for infectious disease prevention and control in MG settings, the primary researcher (NM) and the research team (JA and AV) developed an appraisal tool to assess the quality of the studies. The assessment tool was developed based on published frameworks and public health assessment tools. The questions included in the appraisal tool were divided into 3 categories: study, event, and intervention. The appraisal tool that was developed for this study is presented in Multimedia Appendix 2. A single author (NM) appraised the included studies.

### Data Analysis

The primary researcher (NM) extracted the data using a form developed by the authors to collect relevant data from each article. The form was developed to ascertain the basic features of each study (ie, author and year, study objective, event type, location, duration and year of the event, intervention, aim of the intervention, outcomes, limitations, and recommendations).

## Results

### Study Selection

The database search in Ovid MEDLINE and Embase using the predefined combination of Medical Subject Heading terms yielded 1609 studies from Ovid MEDLINE; 61 (3.79%) were selected for abstract screening, of which 2 (3%) met the eligibility criteria and were selected for inclusion in the review. A total of 82 studies were retrieved from Embase, and 24 (29%) were selected for abstract screening, of which 2 (8%) were included in the analysis after meeting the eligibility criteria.

The database search in CINAHL yielded 71 studies; however, none of the studies met the eligibility criteria after their titles and abstracts were screened, so none were included in the review. The Scopus search (using the advanced search option) yielded 2160 studies; 74 (3.43%) were chosen for title and abstract screening, of which 12 (16%) underwent a full review for potential inclusion and 4 (33%) met the eligibility criteria for inclusion. A draft of the retrieved results is presented in Multimedia Appendix 3.

### Characteristics of the Included Studies

The characteristics of the included studies are summarized in Table 3. The 8 included studies in this review reported MG events in different countries for different purposes where an infectious disease surveillance system was implemented, described, or evaluated. A total of 2 studies on religious gatherings were included: 1 (50%) from Saudi Arabia (the Hajj in 2015 and 2019) [25,27] and 1 (50%) from India (the Prayagraj Kumbh in 2019) [24]. In total, 38% (3/8) of studies on sporting events were included [21,28,29] (the 2014 FIFA World Cup in Brazil, the 2012 Olympic and Paralympic Games in London, and the 2014 Micronesian Games in Pohnpei in the Federated States of Micronesia). A total of 12% (1/8) of studies on a cultural event were included as follows: the 11th Festival of Pacific Arts (FOPA) in the Solomon Islands in 2012 [30]. Only 12% (1/8) of the studies with more than one type of MG event (ie, religious, sporting, and other events) described the surveillance systems used in each event [31]. All the included studies (8/8, 100%) were interventional studies with descriptions of a public health intervention (ie, a surveillance system that was piloted [21], implemented [24,25,27,31], enhanced [29,30], or evaluated [28]).

**Table 3:** Characteristics of the included studies in the review (N=8).

Study, year	Study type	Study objective	Gathering type	Event location and duration	Number of attendees	Attendees’ characteristics
Aggrawal et al [24], 2020	Descriptive	To describe the public health preparedness and implementation of on-site disease surveillance during the Prayagraj Kumbh	Religious	The Prayagraj Kumbh, India January 14, 2019, to March 4, 2019	120 million	Pilgrims Semipermanent residents (comprising sages and pilgrims following different Hindu sects known as akhara sadhus and kalpawasis) who resided at the MG^a^ site for 3 months Traveling pilgrims came for a period of 2-3 days to take a dip in the holy river
Bieh et al [27], 2020	Descriptive analysis	Describe the early warning findings at the Hajj to highlight the pattern of health risks and the potential benefits of the disease surveillance system	Religious	The Hajj Mekkah, Saudi Arabia August 9, 2019, to August 14, 2019	2,489,406 pilgrims 75% international pilgrims	55.6% male attendees 9.2% Saudi nationals
Alotaibi et al [25], 2017	Descriptive	Describe the characteristics of the IDSS^b^ during the 2015 Hajj; highlight best practices and gaps and propose strategies for strengthening and improvement	Religious	The Hajj Mekkah, Saudi Arabia Dhu-AlHijjah 8-14, 2015	1,952,817 pilgrims 193,645 Saudi and 759,172 non-Saudi individuals	Domestic Saudi and non-Saudi pilgrims from 135 countries
Leal Neto et al [21], 2017	Descriptive	Describe the use of a participatory surveillance app for early detection of acute disease outbreaks during the FIFA^c^ World Cup	Sporting	FIFA World Cup, Brazil May 12, 2014, to July 14, 2014	N/A^d^	N/A
White et al [29], 2018	Descriptive	Describe the enhanced surveillance system implemented by Pohnpei State and discuss the sustainable benefits of surveillance of MGs	Sporting	Micronesian Games The Pohnpei Federated State of Micronesia July 19, 2014, to July 29, 2014	1700	Athletes and officials
Hoy et al [30], 2016	Descriptive analysis	Report the experience of providing SS^e^ for the FOPA^f^	Cultural festival	FOPA, Honiara, Solomon Islands July 1, 2012, to July 14, 2012	<3000	Artists and performers
Nsoesie et al [31], 2015	Descriptive	Review and describe novel approaches to disease surveillance	Religious and sporting	The Hajj Kumbh Mela Olympic Games Athletics Super Bowl	N/A	N/A
Severi et al [28], 2014	Descriptive and evaluative	Describe the EBS^g^ implemented by the health protection agency for the London 2012 Olympic and Paralympic Games Evaluate the system’s attributes to identify lessons and contribute knowledge and evidence for planning future MG events	Sporting	London Olympic and Paralympic Games London, United Kingdom July 2, 2012, to September 23, 2012	15,000 athletes, 70,000 volunteers, and 10 million tickets in 11 UK cities	Athletes and volunteers

^a^MG: mass gathering.

^b^IDSS: infectious disease surveillance system.

^c^FIFA: Federation International Football Association.

^d^N/A: not applicable.

^e^SS: syndromic surveillance.

^f^FOPA: Festival of Pacific Arts.

^g^EBS: event-based surveillance.

### Quality of the Included Studies

The quality of the 8 included studies is presented in Multimedia Appendix 4 [21,24,25,27-31]. On the basis of the critical appraisal tool, 50% (4/8) of the studies [21,24,25,31] received low ratings as they scored 10 to 19 points, 38% (3/8) of the studies [27,29,30] received moderate ratings as they scored 20 to 29 points, and 12% (1/8) of the studies [28] received a high rating as they scored 30 to 39 points. We chose not to exclude studies with low ratings from the final synthesis because of the exploratory nature of the review.

### Synthesis of the Results

The results of the included studies were synthesized under 2 themes: type of MG and implementation of the surveillance systems and effectiveness of the digital surveillance systems implemented for infectious disease prevention and control at MG events.

Owing to the different types of MG events included in this review and the variety of surveillance systems identified, the results are reported in 2 tables. Table 3 shows the characteristics of each type of MG event included in the review, the location and duration of the event, and the number and characteristics of the attendees. Table 4 presents the type of surveillance system implemented at each MG event, the aim of the intervention, the outcomes, and further recommendations.

A narrative synthesis was used to report each type of MG event; implementation of the surveillance system; outcomes of the implementation; and the effectiveness, if reported, to summarize the evidence reported in the included studies.

**Table 4:** Characteristics of the surveillance systems implemented at mass gathering (MG) events.

Study, year	Intervention	Aim of the intervention	Result	Outcome	Recommendations
Aggrawal et al [24], 2020	Using standard case definitions, a surveillance framework was developed consisting of an integration of on-site IBS^a^ and EBS^b^ for 22 acute diseases and syndromes Existing disease surveillance program for the town of Prayagraj EI^c^ for the data analysis	To identify and respond to disease outbreaks	95% of reported conditions were communicable diseases, with the most frequently reported being acute respiratory illness, acute fever, and skin infections. The ICC^d^ generated 12 early warning signals for acute diarrhea, thermal events, vector-borne diseases, and vaccine-preventable diseases. 2 outbreaks were reported (chicken pox and a foodborne illness).	On-site disease surveillance provided a public health legacy by successfully implementing an EI-enabled system for early disease detection and response to monitor public health risks.	Investment in disease surveillance as part of public health planning for MGs Developing an ICC with EI capacity for early detection and response Use of a web-based reporting platform Preparedness for the management of respiratory illnesses with epidemic potential Augment the diagnostic capacity of laboratories for outbreaks
Bieh et al [27], 2020	HEWS^e^, used syndromic surveillance and EBS	Rapid detection and response to health threats	The HEWS-generated automated alarms for public health events triggered alerts for rapid epidemiological investigations and facilitated the monitoring of health events. 121 automated alarms were generated for acute febrile syndrome without rash (n=60), severe acute respiratory infections (n=18), acute febrile syndrome with neurological manifestations (n=14), acute febrile syndrome with rash (n=10), heat-related illnesses (n=10), acute jaundice syndrome (n=7), and chemical injuries. 2 events were confirmed by the response team (heat-related illness and injuries and trauma).	The HEWS improved the timeliness of reporting and situational awareness of health events. 2 noninfectious health risks were detected.	Co-operation of countries sending pilgrims to integrate their health clinics with the HEWS database Timely case identification of pilgrims before leaving the health facility
Alotaibi et al [25], 2017	IDSSs^f^ were implemented countrywide in Saudi Arabia for routine facility-based notification of infectious disease events all year round, including during the Hajj An enhanced surveillance system’s tools: HESN^g^ and CITREX^h^	HESN: improve communication among public health professionals involved in outbreak management and provide quality health data for planning and effective allocation of resources CITREX: manage infectious disease data	The IDSSs confirmed 94 cases of malaria, 72 cases of influenza H1N1, a total of 22 cases of food poisoning, 3 cases of dengue fever, and 2 cases of nonmeningococcal meningitis. There were no confirmed cases of MERS-CoV^i^ illness, Ebola virus, or cholera during the 2015 Hajj season.	The enhanced IDSS with electronic tools improved the timeliness of report generation by the public health personnel.	Integrating the existing surveillance data management systems and implementing syndromic surveillance for early warning systems for infectious disease control during the Hajj is necessary International engagement facilitated better disease surveillance
Leal Neto et al [21], 2017	Pilot implementation of a participatory surveillance app (Healthy Cup)	Early detection of acute disease outbreaks	9434 downloads 7177 registered users 4706 active users 47,879 posts 99 posts reported the 10 symptoms	Participatory surveillance through community engagement is an innovative way to conduct epidemiological surveillance. Participatory surveillance has the potential to become a standard component of national health surveillance to improve the early detection of outbreaks and epidemics for timely intervention and risk minimization.	Investment in communication, marketing, and advertising is necessary to penetrate multiple social strata and achieve a broad reach. Investment in digital media to enhance user engagement. The need for reciprocity could ensure continuous engagement with participatory surveillance apps to improve data quality. There is a need for a transparent government role in participatory surveillance.
White et al [29], 2018	ESS^j^ system Web-based SAGES-OE^k^	Early warning and the detection of disease to support the Games	The most common syndrome reported was (ILI^l^; n=225, 55%). Most syndrome cases (75%) were among people from Pohnpei. 7% (30/408) of syndrome cases presented with acute fever and rash, despite the large and ongoing measles outbreak at the time. No new infectious disease outbreak was recorded during the Games.	The enhanced surveillance provided essential data for reassurance of public health security for the Games’ organizers. The SAGES-OE enabled easy entry, storage, collection, and analysis of data and accelerated the SitRep^m^ production because of the simultaneous access to data by multiple users. Regular collection of syndromic surveillance data provides a robust evidence base that can be exploited for better-informed health planning and decision-making.	The need for good planning and preparation, including a substantial lead time of at least 12 months to establish and test web-based surveillance tools for areas with low connectivity and to test methods for the timely manual collection of data The importance of adequate sources of staff to address staff fatigue caused by intense daily surveillance operations over several weeks Effective connections with laboratory services must ensure that the clinical sample collected more closely matches the syndrome’s patterns
Hoy et al [30], 2016	ESS system	To detect and respond to disease outbreaks promptly and effectively Sustain the surveillance system improvements beyond the MG event	A total of 1668 patients presented during the ESS period. The total number of syndrome cases peaked 8 times during the surveillance period.	The ESS provided the necessary elements for detecting and responding to disease outbreaks quickly and effectively.	The sustainability of the ESS can be achieved with adequate lead-in time for engagement and preparation of at least 12 months.
Nsoesie et al [31], 2015	Internet, mobile phone apps, and wireless sensor networks HESN, HAJJ-Mobile decision support system, RODS^n^, MediSys, Healthy Cup, Syndromic Tracking and Reporting System, syndromic surveillance, BioDefend, ESSENCE^o^, Bing, EpiNorth, GPHIN^p^, GeoSentinel Surveillance Network, Twitter, and Wireless and Body Sensor Network	Detect and predict disease outbreaks at some of the world’s largest and most popular MGs	Not reported	HESN: effective during the 2013 Hajj for disease surveillance and monitoring among pilgrims because of the rapid data transmission from practitioners to decision makers	Emerging diagnostic tools can be useful during MGs Rapid point-of-care diagnostic tests for bacterial and viral infections can be useful during MGs
Severi et al [28], 2014	EBS	To detect, validate, analyze, rapidly assess, and report important infectious disease events with potential public health risks that may affect the Games	Sensitivity: EBS: 95.2% ROC^q^: 91.8% PPV^r^: EBS: 32.8% ROC: 77.2% Timeliness EBS timeliness: one day (10th percentile: 0 days-same day; 90th percentile: 3.6 days). DB^s^ timeliness: 2 days (10th percentile: 0 days- same day; 90 percentile; 14.8 days). Acceptability: 96% completeness Stability: able to collect, manage, and provide electronic reports without downtime or system failures Simplicity: good Usefulness: EBS is an efficient information management system that gathers information at local and regional levels in a single collection of data.	The EBS met its objectives.	There is a need to establish specific guidelines for evaluating EI surveillance systems, with attention to the new attributes that better describe these systems’ priorities

^a^IBS: indicator-based surveillance.

^b^EBS: event-based surveillance.

^c^EI: epidemic intelligence.

^d^ICC: incident command center.

^e^HEWS: Health Early Warning System.

^f^IDSS: infectious disease surveillance system.

^g^HESN: Health Electronic Surveillance Network.

^h^CITREX: Electronic Statistical System.

^i^MERS-CoV: Middle East respiratory syndrome coronavirus.

^j^ESS: enhanced syndromic surveillance.

^k^SAGES-OE: Suite for Automated Global Electronic Biosurveillance OpenESSENCE.

^l^ ILI: influenza-like illness.

^m^SitRep: situation report.

^n^RODS: real-time outbreak and disease surveillance.

^o^ESSENCE: Electronic Surveillance System for the Early Notification of Community-Based Epidemics.

^p^GPHIN: Global Public Health Intelligence Network.

^q^ROC: Regional Operation Centres.

^r^PPV: positive predictive value.

^s^DB: HPZone Dashboard.

### Types of MGs and Implemented Surveillance Systems

The characteristics of the surveillance systems implemented at each MG event (religious, sporting, and cultural) are shown in Table 4.

#### Religious MGs

In total, 50% (4/8) of the studies [24,25,27,31] described the implementation of surveillance systems that operated in 2 religious MGs. A total of 75% (3/4) of these studies were related to the Hajj, which is a Muslim religious gathering [25,27,31], and 50% (2/4) were associated with the Kumbh Mela, an Indian Hindu religious gathering [24,31]. Of the 4 studies with religious gatherings, 2 (50%) [24,27] implemented a near–real-time surveillance system for the MG events. The Health Early Warning System (HEWS) consists of syndromic surveillance and EBS, which were implemented in the 2019 Hajj for the rapid detection and response to health threats and helped improve the timeliness of reporting and detecting noninfectious health risks as well as situational awareness of health events [27]. An on-site surveillance system consisted of IBS and EBS, which were implemented at the 2019 Prayagraj Kumbh to identify and respond to disease outbreaks and generate surveillance data, which were useful for public health actions (eg, early detection and response [24]).

In total, 50% (2/4) of the studies [25,31] reported using web-based surveillance systems during the Islamic religious gathering for the Hajj. Alotaibi et al [25] reported the use of 2 electronic web-based surveillance systems that were operational during the 2015 Hajj to strengthen health security: the Health Electronic Surveillance Network (HESN) and the Electronic Statistical System (CITREX). This study reported enhancing the indicator-based infectious disease surveillance system with electronic tools, which improved the timeliness of report generation for public health personnel. Nsoesie et al [31] reported HESN outcomes during the 2013 Hajj in terms of its speed of data transmission from practitioners to decision makers. Nsoesie et al [31] also described the use of a mobile disease support system for the 2009 Hajj for rapid detection, enabling informed decision-making regarding disease control and prevention. However, the outcomes of this system were not reported.

#### Sporting MGs

A total of 50% (4/8) of the studies [21,28,29,31] reported the implementation of surveillance systems in sporting MGs, such as the FIFA World Cup, the Olympic Games, and other (smaller) games.

##### FIFA World Cup

In total, 50% (2/4) of these studies [21,31] described the FIFA World Cup surveillance systems, which included the use of a participatory surveillance system (the Healthy Cup app) that was implemented for the 2014 FIFA World Cup in Brazil for the early detection of acute disease outbreaks. Leal Neto et al [21] reported the advantages of a pilot implementation of participatory surveillance and considered it an essential component of national health surveillance for improving the early detection of outbreaks and epidemics to ensure timely interventions and minimize risk.

Nsoesie et al [31] also described the monitoring system MediSys, which was developed for the 2010 FIFA World Cup in South Africa to enhance epidemic intelligence (EI) activities of collecting information from the internet about potential threats to the public’s health. However, no effective health outcomes were reported in this study.

##### Olympic Games

A total of 75% (3/4) of the studies [28,29,31] described web-based tools that were implemented during several Olympic Games for disease surveillance. White et al [29] reported the outcomes of a web-based enhanced surveillance system known as the Suite for Automated Global Electronic Biosurveillance OpenESSENCE (SAGES-OE). This open-source web-based application was used to support the Eighth Micronesian Games in July 2014 for the early warning and detection of health threats by providing essential data to ensure public health security for the Games’ organizers. The tool’s outcomes showed improved timeliness of data entry, analysis, and dissemination of situation reports (SitReps). Nsoesie et al [31] used a web-based global surveillance system (HealthMap) for infectious diseases before and during the 2010 Vancouver Winter Olympic Games, the 2012 London Summer Olympic Games, and the 2014 Sochi Winter Olympic Games to anticipate threats of disease outbreaks. However, the outcomes of HealthMap were not included in the study. The same study [31] reported the use of a real-time outbreak and disease surveillance system during the 2002 Salt Lake City Winter Olympic Games. The system was set to trigger an alert if unusual disease activity was detected; however, the outcomes of the surveillance system were not reported. A study with outcomes conducted by Severi et al [28] reported the implementation of EBS during the 2012 London Olympic and Paralympic Games to detect, validate, analyze, rapidly assess, and report important infectious disease events and potential public health risks that could have an impact on the Games. The study described EBS as a reliable, reassuring, timely, and simple system, reflecting a stable national EI surveillance tool for the 2021 Games.

##### Other Games

Several syndromic surveillance systems were used to monitor disease outbreaks at the 2001, 2005, and 2007 Super Bowls [31]. The systems were the BioDefend and the Syndromic Tracking and Reporting System. Both systems also tracked bioterrorism and infectious disease threats. The Electronic Surveillance System for the Early Notification of Community-Based Epidemics was another syndromic surveillance system that used data from the emergency department to ensure the early detection of public health events. These systems were widely used to increase situational awareness; however, no health impact was reported.

##### Cultural Festivals

Hoy et al [30] reviewed a surveillance system that was used for the 11th FOPA in the Solomon Islands in 2012. The enhanced syndromic surveillance (ESS) was developed by constructing a web-based tool to enable data entry, data storage, and analysis to detect and respond to disease outbreaks promptly. This study described the system as successful in the early detection of a possible disease outbreak.

### Effectiveness of the Surveillance Systems and the Tools Used for MG Events

#### Overview

The findings of the included studies illustrate the various infectious disease surveillance systems and web-based tools used at the MG events, which provided both passive and active traditional and syndromic surveillance. Several outcomes were reported: better communication, data management, quality of the data, immediate access to the data, real-time data analysis [25], rapid detection of an outbreak [25,30], early warning signs generated by the surveillance data [24,27], improvement in the timeliness of reporting [21,25,27,29,30], and speed of data transmission [31]. The surveillance systems that were described included IBS, EBS, IBS and EBS, syndromic surveillance and EBS, and syndromic surveillance or ESS. The objectives and outcomes are presented in Table 4. The outcomes of the surveillance systems used at each MG event are reported in detail in the following sections.

#### Disease Surveillance for Religious MGs (the Hajj)

The use of disease surveillance for a religious MG (the Hajj) was described in the studies by Alotaibi et al [25] and Bieh et al [27], which reported the implementation outcomes. However, evaluations of the systems were not reported in terms of their effectiveness. Alotaibi et al [25] reported an attempt by the Saudi Ministry of Health (MOH) to strengthen health security during the 2015 Hajj. Owing to infectious disease outbreaks during previous Hajj seasons (eg, the 1865 cholera outbreak and meningococcal disease in 1987, 2000, and 2001), the MOH attempted to improve its surveillance systems by ensuring timely detection of infectious diseases at MG events and minimizing threats to the well-being of pilgrims and their contacts after the MG. An indicator-based infectious disease surveillance system that operated year-round generated and transmitted routine reports from health facilities to the regional and central public health directors of the MOH. During the Hajj, the system was enhanced with 2 electronic surveillance tools (HESN and CITREX) to ensure the timely reporting of event-related information for appropriate action by public health officers. Infectious disease data were collated and entered directly by the hospital surveillance team into the HESN once notification was received from the laboratory, emergency room, isolation ward, or other hospital department in addition to the traditional data updates and reporting tools. An electronic dashboard displayed the uploaded data in the command-and-control center situation rooms. After the data were analyzed, reports were generated in real time and could be accessed immediately by public health officials and decision makers or disseminated through phone messages to responsible persons for immediate action.

In addition, CITREX was only operational during the Hajj, and it complemented HESN in the management of infectious disease data retrieved in real time from the holy areas (Makkah, Medina, Arafat, and Mina). This enhanced system captured data from 3 surveillance teams: the hospital, fixed, and mobile surveillance teams.

The enhanced system was operational from the arrival of the first group of pilgrims (1 month before the Hajj) to the end of the month following the departure of the last group of pilgrims. A list of high-priority infectious diseases during the Hajj was posted with clear guidelines for reporting cases of these diseases.

A 2020 study by Bieh et al [27] described the HEWS that the MOH used to improve the previous system’s limitations at the 2019 Hajj. The system used syndromic surveillance and EBS to detect and respond to potential public health threats during the Hajj. It could alert health authorities to any danger that could lead to a disease outbreak or public health emergency. The HEWS was linked to a public health command control center that mobilized rapid response teams for timely verification and responses to all public health threats. Bieh et al [27] observed a substantial improvement in the speed with which reporting occurred and the detection of 2 noninfectious health risks during the 2019 Hajj, which were attributed to the HEWS.

#### Disease Surveillance for the Kumbh Mela Religious MG

Aggrawal et al [24] reviewed and described a surveillance system implemented for the 2019 Prayagraj Kumbh, with advantages gained after its implementation. However, the system’s effectiveness remains unknown as an evaluation was not conducted.

In total, 2 surveillance systems (IBS and EBS) with EI were implemented to support disease surveillance. EI was used to analyze the data and events, detect signals, verify alerts, and initiate responses. The study reported the performance of the on-site surveillance system that integrated daily IBS and EBS to identify and respond to disease outbreaks. The 2 systems were integrated to enhance the existing Infectious Disease Surveillance Program for the Prayagraj and improve health outcomes. The IBS was implemented to manually and electronically record data from reporting units responsible for the surveillance of patients’ diseases and mortality and to monitor reports of water quality. The EBS helped identify health-related events based on preidentified triggers from the media, private health facilities, and food safety departments. Integrating the IBS and EBS systems with the existing Infectious Disease Surveillance Program provided surveillance data that were valid for the early detection of disease and the response required for public health action. The study found multiple outcomes that were beneficial for the successful implementation of EI for the early detection and rapid response to public health risks.

#### Disease Surveillance Systems Implemented for Sporting MGs (FIFA World Cup, Olympic Games, Micronesian Games, and FOPA)

Leal Neto et al [21] described the implementation of participatory surveillance for the FIFA World Cup in Brazil in 2014 (Healthy Cup). The app was designed for the early detection of acute disease outbreaks during the MG event. In total, 3 primary infectious disease outcomes related to sporting MGs were the focus of the surveillance: respiratory syndromes (influenza, measles, and rubella), diarrhea (acute diarrhea and cholera), and rash syndromes (dengue fever). Respiratory syndrome occurred with the greatest frequency based on the reports generated by the Healthy Cup app.

This study shed light on the inexpensive advantage of participatory surveillance for the timely acquisition of records and the sharing of data, as well as the platform’s scalability and capacity for integration with the population served. The surveillance system’s effectiveness is unknown as an evaluation was not conducted.

The SAGES-OE ESS system, described by White et al [29] as a customized web-based application for the 2014 Micronesian Games, was implemented for the early warning and detection of disease. The surveillance was operational for 21 days, from July 17 (2 days before the Games) until August 6 (1 week after the Games). A total of 11 sentinel sites and 8 disease syndromes were selected for surveillance: acute fever and rash, influenza-like illness, prolonged fever, fever and jaundice, watery diarrhea, nonwatery diarrhea, foodborne disease outbreaks, and heat-related illness. Surveillance data on the number of acute care encounters and syndrome cases were collected manually at the sentinel sites and entered into the web-based SAGES-OE. Daily SitReps of the epidemiological situation were summarized using the analytical and visualization tools of the SAGES-OE.

This study examined the importance of web-based technology as a key feature of enhanced surveillance. The findings included improvements in the timely entry, storage, collection, and analysis of data, which facilitated the use of SitReps by key stakeholders and decision makers. The study also described the SAGES-OE as a constructive model for the surveillance of future MGs across the Pacific [29]. However, the effectiveness of the system could not be determined.

Severi et al [28] described and evaluated the enhancement of the existing IBS system with a new surveillance approach. The EBS was implemented from July 2, 2012, to September 23, 2012, a total of 2 weeks before the beginning of the Paralympic Games until 2 weeks after the end of the Games. The EBS was implemented as a safety net for the detection, validation, analyses, and rapid assessment and reporting of actual and potentially important infectious disease outbreaks at the London 2012 Olympic and Paralympic Games. The study reported the effectiveness of the EBS in meeting its objectives and key attributes in the context of an MG, including its usefulness, reliability, stability, and acceptability as a reporting system that met the daily reporting and reassurance needs of the Olympic Coordination Centre. The effectiveness of the system was measured based on several attributes: timeliness, sensitivity, positive predictive value, completeness, usefulness, acceptability, simplicity, and stability.

Hoy et al [30] reported the outcomes of a developed web-based system that was operated during the 11th FOPA in 2012. The ESS was designed to enable data entry, storage, and analysis to detect possible infectious disease outbreaks. The outcomes reported were the early detection of disease outbreaks and public health responses to them. However, effectiveness was not reported.

## Discussion

### Principal Findings

The objectives of this review were to (1) identify the different public health digital surveillance systems implemented at MG events for infectious disease prevention and control and (2) assess the evidence on the effectiveness of digital surveillance systems implemented for infectious disease prevention and control at MG events.

We found that the most common types of surveillance systems used in MG events, either independently or with other systems, varied among IBS; EBS; syndromic surveillance; or the use of EI, which consists of IBS and EBS. Of the 8 studies included in this review, 7 described different surveillance systems and web-based tools that were implemented at MG events for infectious disease prevention and control. They reported beneficial outcomes of implementation [21,24,25,27,29-31] (Table 4). Only one study described and evaluated an implemented surveillance system and reported its effectiveness based on attributes relevant to the context of MGs that were measured (sensitivity, positive predictive value, timeliness, acceptability, stability, simplicity, and usefulness) [28]. However, these 7 attributes were not fully reported or measured in 88% (7/8) of the studies [21,24,25,27,29-31] (Table 5). Each attribute is defined in Multimedia Appendix 5 [32]. With only 1 comprehensive report [28] of the evaluation of the implemented surveillance system, there is limited evidence of the effectiveness of public health digital surveillance systems for infectious disease prevention and control at MG events.

**Table 5:** Reported attributes indicating the effectiveness of the surveillance systems implemented in the included studies.

Study, year	Attribute								Evidence of effectiveness
	Sensitivity	PPV^a^	Timelines	Acceptability	Stability	Simplicity	Usefulness		
Aggrawal et al [24], 2020	NR^b^	NR	NR	NR	NR	NR	R^c^	Effectiveness cannot be determined	
Bieh et al [27], 2020	R	NR	R	NR	NR	NR	NR	Effectiveness cannot be determined	
Alotaibi et al [25], 2017	NR	NR	R	NR	NR	NR	NR	Effectiveness cannot be determined	
Leal Neto et al [21], 2017	R	NR	R	NR	NR	NR	NR	Effectiveness cannot be determined	
White et al [29], 2018	R	NR	R	NR	NR	NR	NR	Effectiveness cannot be determined	
Hoy et al [30], 2016	NR	NR	R	NR	NR	R	NR	Effectiveness cannot be determined	
Nsoesie et al [31], 2015	NR	NR	NR	NR	NR	NR	NR	Effectiveness cannot be determined	
Severi et al [28], 2014	RM^d^	RM	RM	RM	RM	RM	RM	Effective based on the evaluation	

^a^PPV: positive predictive value.

^b^NR: not reported.

^c^R: reported.

^d^RM: reported and measured.

### Strengths of the Surveillance Systems

The real-time on-site surveillance systems that operated during the MG events illustrated their usefulness in generating valid surveillance data for public health actions that were based on early detection [24]. Although the ability to detect infectious hazards was important during the MGs, the real-time surveillance systems also improved the detection and timeliness of reporting noninfectious health risks during the 2019 Hajj [27].

The enhancement of traditional passive surveillance systems with real-time active surveillance, consisting of IBS with EBS or syndromic surveillance with EBS, proved useful. Early warning signs are essential in MGs for the early detection of disease and timely response to this warning to mitigate the risk of disease spread and outbreaks. These warnings are provided with real-time reporting of both syndromic surveillance and EBS data. The combination of syndromic and EBS complements case-based surveillance systems in MGs. Syndromic surveillance is characterized by the rapid detection of health threats and the response to them, with improvements in emerging disease surveillance. In contrast, EBS triggers public health alerts by collecting unstructured data from various settings through a prediagnostic data analysis. Health care facilities, the media, and the community are examples of settings where event data are collected [27].

Syndromic surveillance data were found to be a powerful tool that, if used efficiently, can better inform health planning and decision-making for purposes other than outbreak detection [29]. In addition, ESS can contribute to the early detection of diseases [30]. Compared with traditional EBS, EBS as a component of EI is different and was considered an adequate safety net that was reliable, reassuring, timely, simple, and stable for the London 2012 Olympic Games [28]. Participatory surveillance provides comprehensive access to a range of data from users worldwide. It involves benefits at a lower cost, timely data acquisition, information collection and sharing, platform scalability, and the capacity for integration between the population being served and public health services [21].

### Challenges of the Surveillance Tools

Owing to data extraction and response limitations, the HEWS operated as a near–real-time surveillance system. In a congested MG such as the Hajj where attendees (pilgrims) are mobile, it is crucial to have immediate case identification for better decision-making [27]. In the case of Kumbh Mela, the absence of baseline data did not permit the generation of outbreak thresholds from surveillance data [24].

Mistakes in classification and missing surveillance data because of the closure of health facilities on weekends during the art festival led to potential bias in the surveillance system. Untrained laboratory staff led to miscommunication with the clinical staff at the sentinel sites responsible for responding to outbreaks [30].

For participatory surveillance, apps such as the Healthy Cup might be an effective tool to identify potential threats of outbreaks associated with MGs. However, the attendees’ engagement during the event was low because of exposure bias to the app depending on the users’ age and language barriers restricting app use [21].

### Importance of Evaluations and Recommendations for Further Research

Surveillance systems vary in their methods, scope, and objectives [33]. Evaluation of surveillance systems is essential to assess whether the system is meeting its objectives and effectively using available resources [34]. There is limited information on the usefulness of surveillance systems for infectious disease control and outbreak detection [32]. Guidelines for evaluating public health surveillance systems have existed since 1988, with updates by the Centers for Disease Control and Prevention and a framework for evaluating public health surveillance systems for the early detection of outbreaks [32]. Nevertheless, there are no international standards or guidelines for evaluating digital public health surveillance systems for infectious disease prevention and control with a focus on the MG context.

An evaluation of the EBS system implemented at the 2012 London Olympic and Paralympic Games [28] was conducted using the *Updated Guidelines for Evaluating Public Health Surveillance Systems* from the US Centers for Disease Control and Prevention. A selection of attributes relevant to sporting MG events was reported in a study [28] to assess the system that was implemented based on the sporting context. According to one study [34], some of the systems’ attributes were more critical for selected surveillance purposes and health outcomes than others. This finding indicates that the system-chosen attributes used in the London Games [28] were important and relevant to the nature of the MG event.

In this review, the surveillance systems for the MG events were implemented for similar reasons. The primary and essential aim of the implemented surveillance systems during the MG events was to provide early warning of health events and to detect, identify, and respond to disease outbreaks rapidly. Several studies included in this review (5/8, 62%) reported a vital attribute achieved after implementing or enhancing the existing surveillance system, namely, an improvement in the timeliness of reporting and data sharing [21,25,27,29,30]. However, a precise measurement of this attribute and its assessment were not reported or explained. Timeliness is a key performance measure of public health surveillance systems [35]. Periodic evaluation of such attributes is crucial as it reflects time delays between all the response steps in the public health surveillance process. Severi et al [28] measured the effectiveness of the reported timelines in terms of the time between the entry of a new event into the health protection agency “HP” zone (an electronic public health case management tool used by a local health protection unit) and the time of its report to the EBS. Timeliness is critical when data are to be used to implement immediate disease control and prevention activities for infectious diseases that are acute, severe, and highly transmissible [35].

Disease-specific surveillance, which is the traditional type of surveillance, has existed for many years and was the chief component of public health strategies [14]. This type of surveillance data is considered accurate [36]; however, they do not identify emerging infectious diseases because of limited resources, time, and reporting systems [16]. Timeliness and sensitivity are the most essential features of surveillance systems to detect outbreaks and identify health threats early. The WHO 2005 International Health Regulations [9] emphasize the importance of enhancing the existing web-based tools of surveillance systems to complement traditional passive surveillance systems with active surveillance systems to improve the timeliness and accuracy of the existing surveillance capacity. The enhancement of existing surveillance systems with nontraditional systems, such as EBS, syndromic surveillance, or digital surveillance, can improve the timeliness of reporting. These systems are high in sensitivity but low in specificity compared with traditional surveillance systems [3], and these new systems cannot be considered alternatives or replacements for traditional surveillance systems. However, they are an extension that complements and reinforces the capacity of traditional systems [18]. This characteristic was illustrated in the study by Aggrawal et al [24], where a combination of the 2 types of surveillance was used to complement each other: EI (IBS and EBS).

The review and evaluation of surveillance systems are critical for improving their performance and cost-effectiveness [33]. They enable the continuous improvement of data and system quality. However, with only 12% (1/8) of the studies reporting an evaluation of public health digital surveillance systems for infectious disease prevention and control at MGs, the effectiveness of these systems cannot be determined.

This review highlights the need to develop a comprehensive approach to evaluating digital public health surveillance systems for infectious disease prevention and control at MG events. A comprehensive evaluation approach can be achieved by targeting multiple aspects of digital surveillance systems’ characteristics. This process can begin before deployment by applying a systems engineering approach to identify the challenges of surveillance system implementation and effectiveness. The assessment should cover the social and economic aspects of the intervention or system features in addition to the epidemiological aspects. Simulations could be used to identify the key performance characteristics of the components of the system that contribute the most to overall effectiveness. This approach will provide a complete understanding of the multiple factors and constraints that stakeholders need to consider when implementing a digital surveillance system for an MG event. It will highlight the key determinants for the successful implementation of disease surveillance systems, including effectiveness and cost-effectiveness. It is recommended that the design of such an evaluation be flexible to the context of the surveillance systems as well as operational.

### Implications for Research

This review’s findings revealed limited evidence for the availability of standard international guidelines for evaluating public health digital surveillance systems for infectious disease prevention and control in the MG context. Moreover, most studies (7/8, 88%) did not report evaluations of digital public health surveillance systems for infectious disease prevention and control that were implemented at MG events.

Another standardized guideline or framework is needed to evaluate digital surveillance systems in MGs for infectious disease prevention and control.

Additional reporting guidelines for interventional studies should provide a minimum amount of information needed to report the implementation of digital public health surveillance systems at MG events. This will facilitate the reporting by surveillance systems at MGs and increase the number of evaluation studies.

A quality assessment tool is necessary for interventional studies that evaluate digital public health surveillance systems for infectious disease prevention and control at MGs. This tool will facilitate the assessment of interventional studies’ reports of surveillance systems at MG events.

The perspectives of stakeholders of MGs on the performance of existing surveillance systems and their intentions to evaluate digital public health surveillance systems should be investigated.

### Strengths and Limitations of This Systematic Review

This research is the first systematic review that highlights the gap in the literature on evidence related to the effectiveness of digital public health surveillance systems implemented at MGs for infectious disease prevention and control and the need to establish a standard guideline for such evaluations. Given the increase in emerging infectious diseases such as COVID-19, which affected countries and MG events worldwide, new technologies and approaches to detection have been developed. Therefore, evaluating surveillance systems and new digital technologies implemented at MG events for infectious disease prevention and control is crucial for measuring their effectiveness. We developed a new appraisal tool for this review, but it has not been validated.

This study has several limitations. First, only 4 databases were searched. Although these 4 databases are the most relevant sources of health informatics publications, it is possible that relevant studies were missed. Although gray literature would have retrieved studies that might have met the eligibility criteria, it was not included because of the absence of peer-reviewed studies. Second, the screening of the titles and abstracts of the studies retrieved from the databases was performed by a single author. However, we believe that this did not affect the quality of this study. Furthermore, ratings of the quality of the included studies based on the developed appraisal tool varied between low and moderate because of the absence of studies reporting evaluations of the surveillance systems implemented at MGs. The number of studies included in this review might be considered low because of the limited number of studies that met the eligibility criteria of studies evaluating digital surveillance systems for infectious disease prevention and control at MG events to measure effectiveness. However, we believe that these limitations do not affect the validity of our findings.

### Conclusions

Effective and timely communicable disease control relies on effective and timely disease surveillance. The nature of emerging infectious diseases often limits the effectiveness of traditional surveillance systems. Digital surveillance could improve the sensitivity and timeliness of the detectors of health events and facilitate responses to emerging diseases [18]. Hence, the risk of transmission of infectious diseases during MG events remains.

There is sparse evidence of public health digital surveillance systems as an effective method for infectious disease prevention and control at MG events owing to the limited number of evaluation studies.

This finding indicates a methodological challenge in evaluating surveillance systems at MGs because of a lack of available guidelines for evaluating digital surveillance systems for infectious disease prevention and control at MG events. Hence, further research on the effectiveness of public health digital surveillance systems for infectious disease prevention and control is necessary.

## Appendix

Multimedia Appendix 1 Search strategies used in the Ovid MEDLINE, Embase, CINAHL, and Scopus databases.

Multimedia Appendix 2 Critical appraisal tool for interventional public health digital surveillance systems studies.

Multimedia Appendix 3 Results of the search strategies used in Ovid MEDLINE, Embase, and Scopus Query.

Multimedia Appendix 4 Quality assessment of the included studies using the developed appraisal tool for interventional public health digital surveillance systems studies.

Multimedia Appendix 5 Description of surveillance system attributes.

## Abbreviations

CITREX: Electronic Statistical System
EBS: event-based surveillance
EI: epidemic intelligence
ESS: enhanced syndromic surveillance
FIFA: Federation International Football Association
FOPA: Festival of Pacific Arts
HESN: Health Electronic Surveillance Network
HEWS: Health Early Warning System
IBS: indicator-based surveillance
MG: mass gathering
MOH: Saudi Ministry of Health
PRISMA: Preferred Reporting Items for Systematic Reviews and Meta-Analyses
SAGES-OE: Suite for Automated Global Electronic Biosurveillance OpenESSENCE
SARS: severe acute respiratory syndrome
SitRep: situation report
WHO: World Health Organization
