# Supplementary material for: Effectiveness of Public Health Digital Surveillance Systems for Infectious Disease Prevention and Control at Mass Gatherings: Systematic Review
Source: J Med Internet Res. 2023 May 19;25:e44649. doi: 10.2196/44649 (PMC10238952; doi:10.2196/44649)
Supplement: Multimedia Appendix 1 [file jmir_v25i1e44649_app1.docx]

## Multimedia Appendix 1

Subject headings (MeSH) terms and keywords used in the search of Ovid MEDLINE, CINAHL, Embase, and Scoups.

(A8, B8, C11, D15) was used in searches of Ovid MEDLIE, Embase and CINAHL

(A5, B8, C9, D14) was used in searches of Scoups

Ovid MEDLINE, Embase search strategy:

Category A: Population (Event)

1. Mass Gatherings
2. Mass Event
3. Large Gathering
4. Large Events
5. Crowding
6. Religious Gatherings
7. Hajj
8. 1 or 2 or 3 or 4 or 5 or 6 or 7

Category B: Phenomenon of Interest (Public Health)

1. Public Health
2. Health Protection
3. Health Prevention
4. Prevention & Control
5. Public Health Surveillance
6. Emergency Preparedness
7. Mass Gatherings Preparedness
8. 1 or 2 or 3 or 4 or 5 or 6 or 7

Category C: Focus (Infectious Disease)

1. Infectious Disease
2. Communicable Disease
3. Infectious Disease Control
4. Communicable Disease Control
5. Infectious Disease Prevention
6. Communicable Disease Prevention
7. Infectious Disease Surveillance
8. Communicable Disease Surveillance
9. Outbreaks
10. Pandemic
11. 1 or 2 or 3 or 4 or 5 or 6 or 7 or 8 or 9 or 10

Category D: Instrument (Technology)

1. Digital Health
2. Digital Technolgies
3. Digital Tools
4. Digital interventions
5. Digital Solutions
6. Detection
7. Prediction
8. Simulation
9. Testing
10. Forecasting
11. Tracking and Tracing
12. Modelling
13. Artificial Intelligence
14. Machin Learning
15. 1 or 2 or 3 or 4 or 5 or 6 or 7 or 8 or 9 or 10 or 11 or 12 or 13 or 14

Scoups search strategy and terms:

Category A: Population (Event)

1. Mass Gatherings
2. Mass Event
3. Religious Gathering
4. Hajj
5. 1 or 2 or 3 or 4

Category B: Phenomenon of Interest (Public Health)

1. Public Health
2. Health Protection
3. Health Prevention
4. Prevention & Control
5. Public Health Surveillance
6. Emergency Preparedness
7. Mass Gathering Preparedness
8. 1 or 2 or 3 or 4 or 5 or 6 or 7

Category C: Focus (Infectious Disease)

1. Infectious Disease
2. Communicable Disease
3. Infectious Disease Control
4. Communicable Disease Control
5. Infectious Disease Prevention
6. Communicable Disease Prevention
7. Infectious Disease Surveillance
8. Communicable Disease Surveillance
9. 1 or 2 or 3 or 4 or 5 or 6 or 7 or 8

Category D: Instrument (Technology)

1. Digital Health
2. Digital Technolgies
3. Digital Tools
4. Digital interventions
5. Digital Solutions
6. Detection
7. Prediction
8. Simulation
9. Forecasting
10. Modelling
11. Artificial Intelligence
12. Machin Learning
13. 1 or 2 or 3 or 4 or 5 or 6 or 7 or 8 or 9 or 10 or 11 or 12
