# Supplementary material for: Effectiveness of Public Health Digital Surveillance Systems for Infectious Disease Prevention and Control at Mass Gatherings: Systematic Review
Source: J Med Internet Res. 2023 May 19;25:e44649. doi: 10.2196/44649 (PMC10238952; doi:10.2196/44649)
Supplement: Multimedia Appendix 2 [file jmir_v25i1e44649_app2.docx]

## Multimedia Appendix 2

**Critical Appraisal Tool for Interventional Public Health Digital Surveillance Systems Studies**

***Study***

1. Is it an interventional study? Yes/No
2. Is the study type appropriate for the research question/ objective? Yes/No
3. Is the study Descriptive, Evaluative, or Both? (Circle one)
4. Is the objective of the study stated clearly? Yes/No
5. Did the study follow a reporting guideline? Yes/No
6. Did the study state the aim of the intervention? Yes/No
7. Did the study report the outcomes of the intervention? Yes/No
8. Did the study report effectiveness of intervention outcomes? Yes/No
9. Have the results been interpreted appropriately? Yes/No
10. Are there any recommendations in the study for further intervention improvements? Yes/No

***Event:***

1. Was the event described clearly?
2. Type of the Event Yes/No
3. Identified Audience Yes/No
4. Number of attendees Yes/No
5. Duration of the Event Yes/No

***Intervention:***

1. Was the surveillance system defined/described? Yes/No
2. Was the objective of the surveillance system defined clearly? Yes/No
3. Was the performance of the surveillance system measured? Yes/No
4. How many system attributes were reported? ( )
5. What were they? ( )
6. Were the systems' attributes evaluated after the report? Yes/No
7. Did the aim of the intervention meet with the outcomes? Yes/No
8. Was the system piloted or implemented? ( )
9. Were disease syndromes described? Yes/No

*Scoring key for the assessment of the levels of quality of the included studies: low, moderate, or high.*

*Low: 10-19 Moderate: 20-29 High: 30-39*

Yes = 1; No = 0

Descriptive = 0; Evaluative = 1

Piloted = 1; Implemented = 2

Number of Attributes = a score for each attribute was reported.

System Attributes: Timelines and sensitivity scores =2; each/all other attributes' scores =1.
