# Supplementary material for: Effectiveness of Public Health Digital Surveillance Systems for Infectious Disease Prevention and Control at Mass Gatherings: Systematic Review
Source: J Med Internet Res. 2023 May 19;25:e44649. doi: 10.2196/44649 (PMC10238952; doi:10.2196/44649)
Supplement: Multimedia Appendix 3 [file jmir_v25i1e44649_app3.docx]

## Multimedia Appendix 3

**Ovid MEDLINE(R) retrieved results**

1 Mass Gathering.mp. 532

2 crowding/ 3725

3 disaster planning/ 15439

4 Mass Gatherings.mp. 615

5 Mass Events.mp. 58

6 Large Gathering.mp. 26

7 pandemic/ or epidemics/ 110841

8 "crowding (area)"/ 0

9 crowding.mp. 12466

10 1 or 2 or 3 or 4 or 5 or 6 or 7 or 8 or 9 138453

11 religious mass gatherings.mp. 16

12 public health/ or epidemics/ 105198

13 Hajj.mp. 648

14 Saudi Arabia/ 16387

15 religious mass gathering.mp. 13

16 11 or 12 or 13 or 14 or 15 121661

17 10 or 16 241943

18 Public Health.mp. 348919

19 public health/ 92426

20 Health Protection.mp. 3750

21 Health Prevention.mp. 1501

22 public health/ or prevention/ 92426

23 (Prevention and Control).mp. [mp=title, book title, abstract, original title, name of substance word, subject heading word, floating sub-heading word, keyword heading word, organism supplementary concept word, protocol supplementary concept word, rare disease supplementary concept word, unique identifier, synonyms] 1506975

24 Public Health Surveillance.mp. 7203

25 public health surveillance/ 5191

26 Emergency Preparedness.mp. 2111

27 Mass Gathering Preparedness.mp. 5

28 18 or 19 or 20 or 21 or 22 or 23 or 24 or 25 or 26 or 27 1789968

29 infectious disease.mp. 63751

30 communicable disease.mp. 33876

31 communicable disease/ 32479

32 infectious disease control.mp. 553

33 infection control/ 28487

34 communicable disease control.mp. 30372

35 communicable disease control/ 30020

36 infectious disease prevention.mp. 226

37 infection prevention/ 0

38 communicable disease prevention.mp. 141

39 outbreaks.mp. 119154

40 epidemics/ 13321

41 pandemic.mp. 110574

42 infectious disease surveillance.mp. 505

43 disease surveillance/ 0

44 public health/ 92426

45 Public Health Surveillance.mp. 7203

46 public health surveillance/ 5191

47 communicable disease surveillance.mp. 380

48 29 or 30 or 31 or 32 or 33 or 34 or 35 or 36 or 37 or 38 or 39 or 40 or 41 or 42 or 43 or 44 or 45 or 46 or 47 439028

49 Digital Health.mp. 3581

50 Digital Technologies.mp. 1320

51 Digital Technology.mp. 2012

52 digital technology/ 525

53 Digital Interventions.mp. 477

54 digital intervention.mp. 325

55 digital solution.mp. 68

56 disease detection.mp. 3117

57 digital disease detection.mp. 27

58 prediction.mp. 264952

59 detection.mp. 914076

60 simulation.mp. 380960

61 forecasting.mp. 96456

62 "prediction and forecasting"/ 0

63 modelling.mp. 84154

64 testing.mp. 689503

65 (tracking and tracing).mp. [mp=title, book title, abstract, original title, name of substance word, subject heading word, floating sub-heading word, keyword heading word, organism supplementary concept word, protocol supplementary concept word, rare disease supplementary concept word, unique identifier, synonyms] 1039

66 49 or 50 or 51 or 52 or 53 or 54 or 55 or 56 or 57 or 58 or 59 or 60 or 61 or 62 or 63 or 64 or 65 2255973

67 Artificial Intelligence.mp. 39132

68 artificial intelligence/ 34078

69 Machine Learning.mp. 53333

70 machine learning/ 28240

71 67 or 68 or 69 or 70 83915

72 10 and 28 and 48 and 66 5730

73 66 or 71 2300936

74 10 and 28 and 48 and 73 5870

75 17 and 28 and 48 and 73 11976

76 16 and 28 and 48 and 73 7749

77 16 and 72 1609

**Embase retrieved results**

1 Mass Gatherings.mp. 707

2 Crowding.mp. 18815

3 Mass Events.mp. 90

4 Large events.mp. 158

5 Large Gatherings.mp. 98

6 Religious Gathering.mp. 30

7 Hajj.mp. 980

8 1 or 2 or 3 or 4 or 5 or 6 or 7 20524

9 Public Health.mp. 543130

10 Health Protection.mp. 4954

11 Health Prevention.mp. 2174

12 Public Health surveillance.mp. 3444

13 Emergency Preparedness.mp. 2953

14 9 or 10 or 11 or 12 or 13 549690

15 infectious Disease.mp. 58584

16 Communicable Disease.mp. 50200

17 Infectious disease control.mp. 752

18 communicable disease control.mp. 4284

19 infectious disease prevention.mp. 291

20 communicable disease prevention.mp. 207

21 outbreaks.mp. 67337

22 pandemic.mp. 212599

23 infectious disease surveillance.mp. 636

24 communicable disease surveillance.mp. 394

25 15 or 16 or 17 or 18 or 19 or 20 or 21 or 22 or 23 or 24 361625

26 Digital Health.mp. 5920

27 Digital Technologies.mp. 2142

28 Digital Technology.mp. 4677

29 Digital Interventions.mp. 750

30 Digital Solutions.mp. 444

31 prediction.mp. 721086

32 detection.mp. 1461941

33 simulation.mp. 506925

34 forecasting.mp. 74761

35 testing.mp. 1065544

36 (tracking and tracing).mp. [mp=title, abstract, heading word, drug trade name, original title, device manufacturer, drug manufacturer, device trade name, keyword heading word, floating subheading word, candidate term word] 1506

37 Modelling.mp. 135918

38 Artificial intelligence.mp. 53976

39 Machine Learning.mp. 105389

40 26 or 27 or 28 or 29 or 30 or 31 or 32 or 33 or 34 or 35 or 36 or 37 or 38 or 39 3712392

41 8 and 14 and 25 and 40 83

**Scopus query and retrieved results**

2,160 document results

( ( "Mass Gatherings" )  OR  ( "Mass Events" )  OR  ( "Religious Gathering" )  OR  ( "Hajj" ) )  AND  ( ( "Public Health" )  OR  ( "Health Protection" )  OR  ( "Health Prevention" )  OR  ( "Prevention & Control" )  OR  ( "Public Health Surveillance" )  OR  ( "Emergency Preparedness" )  OR  ( "Mass Gathering Preparedness" ) )  AND  ( ( "Infectious Disease" )  OR  ( "Infectious Disease Control" )  OR  ( "Infectious Disease Prevention" )  OR  ( "Infectious Disease Surveillance" )  OR  ( "Communicable Disease" )  OR  ( "Communicable Disease Control" )  OR  ( "Communicable Disease Prevention" )  OR  ( "Communicable Disease Surveillance" ) )  AND  ( ( "Digital Health" )  OR  ( "Digital Technology" )  OR  ( "Digital Interventions" )  OR  ( "Digital Tools" )  OR  ( "Digital Solutions" )  OR  ( "Digital Disease Detection" )  OR  ( "Prediction" )  OR  ( "Simulation" )  OR  ( "Forecasting" )  OR  ( "Modeling" )  OR  ( "Artificial Intelligence" )  OR  ( "Machine Learning" ) )
