# Supplementary material for: Effectiveness of Public Health Digital Surveillance Systems for Infectious Disease Prevention and Control at Mass Gatherings: Systematic Review
Source: J Med Internet Res. 2023 May 19;25:e44649. doi: 10.2196/44649 (PMC10238952; doi:10.2196/44649)
Supplement: Multimedia Appendix 4 [file jmir_v25i1e44649_app4.docx]

## Multimedia Appendix 4

Table S1 Quality assessment for the included studies in the review

| References in alphabetical order | Study | | | | | | | | | | | Event | | | | | Intervention | | | | | | | | | | Score | | Quality of the Study | |
| --- | --- | --- | --- | --- | --- | --- | --- | --- | --- | --- | --- | --- | --- | --- | --- | --- | --- | --- | --- | --- | --- | --- | --- | --- | --- | --- | --- | --- | --- | --- |
|  | 1 | 2 | 3 | 4 | 5 | 6 | 7 | 8 | 9 | 10 | 11.a | | 11.b | 11.c | 11.d | 12 | | 13 | 14 | 15 | 16 | 17 | 18 | 19 | 20 |  | |  | |  |
| Aggrawal et al. 2020 (24) | Y | Y | D | Y | N | Y | Y | N | Y | Y | Y | | Y | Y | Y | Y | | Y | N | 1 | Usefulness | N | Y | I | Y | 19 | | Low | |  |
| Alotaibi et al. 2017 (25) | Y | Y | D | Y | N | Y | Y | N | Y | Y | Y | | Y | Y | Y | Y | | Y | N | 1 | Timeliness | N | Y | I | N | 19 | | Low | |  |
| Bieh et al. 2020 (27) | Y | Y | D | Y | N | Y | Y | N | Y | Y | Y | | Y | Y | Y | Y | | Y | N | 2 | Timeliness Sensitivity | N | Y | I | Y | 23 | | Moderate | |  |
| Hoy et al. 2016 (30) | Y | Y | D | Y | N | Y | Y | N | Y | Y | Y | | Y | Y | Y | Y | | Y | N | 2 | Timeliness  Simplicity | N | Y | I | Y | 23 | | Moderate | |  |
| Neto et al. 2017 (21) | Y | Y | D | Y | N | Y | Y | N | Y | Y | Y | | N | N | Y | Y | | Y | N | 2 | Timeliness Sensitivity | N | Y | P | Y | 19 | | Low | |  |
| Nsoesie et al. 2015 (31) | Y | Y | D | Y | N | Y | Y | N | Y | Y | Y | | N | N | N | Y | | Y | N | 0 | Not reported | N | Y | I | N | 13 | | Low | |  |
| Severi et al. 2014 (28) | Y | Y | D&  E | Y | N | Y | Y | Y | Y | Y | Y | | Y | Y | Y | Y | | Y | Y | 7 | Sensitivity  PPV  Timeliness  Acceptability  Stability  Simplicity  Usefulness | Y | Y | I | Y | 37 | | High | |  |
| White et al. 2018 (29) | Y | Y | D | Y | N | Y | Y | N | Y | Y | Y | | Y | Y | Y | Y | | Y | N | 2 | Timeliness  Sensitivity | N | Y | I | Y | 23 | | Moderate | |  |
