# Supplementary material for: Effectiveness of Public Health Digital Surveillance Systems for Infectious Disease Prevention and Control at Mass Gatherings: Systematic Review
Source: J Med Internet Res. 2023 May 19;25:e44649. doi: 10.2196/44649 (PMC10238952; doi:10.2196/44649)
Supplement: Multimedia Appendix 5 [file jmir_v25i1e44649_app5.docx]

## Multimedia Appendix 5

Table S2 Description of surveillance systems attributes (Klaucke et al., 1988)

| Attribute | Definitions |
| --- | --- |
| Sensitivity | The sensitivity of a surveillance system can be considered on two levels. First, at the level of case reporting, sensitivity refers to the proportion of cases of a disease (or other health-related event) detected by the surveillance system. Second, sensitivity can refer to the ability to detect outbreaks and monitor changes in the number of cases over time. |
| Positive predictive value | Predictive value positive (PVP) is the proportion of reported cases that have the health-related event under surveillance. |
| Timeliness | Timeliness reflects the speed between steps in a public health surveillance system. |
| Acceptability | Acceptability reflects the willingness of persons and organisations to participate in the surveillance system. |
| Stability | Stability refers to the reliability (i.e., the ability to collect, manage, and provide data properly without failure) and availability (the ability to be operational when needed) of the public health surveillance system. |
| Simplicity | A public health surveillance system's simplicity refers to its structure and ease of operation. Surveillance systems should be as simple as possible while still meeting their objectives. |
| Usefulness | A public health surveillance system is useful if it contributes to preventing and controlling adverse health-related events, including an improved understanding of the public health implications of such events. A public health surveillance system can also be useful if it helps to determine that an adverse health-related event previously thought to be unimportant is essential. In addition, data from a surveillance system can be useful in contributing to performance measures, including health indicators that are used in needs assessments and accountability systems. |
